# Gonorrhea: Its Treatment by Intravesical Injections of Potassium Permanganate

**Published:** 1895-10

**Authors:** 


### Gonorrhea: Its Treatment by Intravesical injections of Potassium Permanganate.

Since my return from Europe, on April 19, I have satisfactorily employed, with but slight modifications, the treatment of gonorrhea introduced by Prof. Janet, of Paris. Most of these cases were in my department at the West Side German Dispensary, and thus far seem to bear out the results obtained in Europe.

Simple and effective as the treatment is, it is equally surprising that as yet it appears to be but little known in our country.

I sketch it here, as well as I am able to do in words, with the hope that others may employ it and favor me with their results, to be used in a more extended study of the subject, shortly to be published.

The apparatus employed consists essentially of a glass irrigator capable of holding two thousand grammes; to this is attached a rubber tube three hundred centimetres (about one hundred and twenty inches) long, whose free end is slipped over a glass nozzle about seven centimetres long and six centimetres in circumference, running to a blunt point, which can easily be pressed into the meatus to occlude it entirely. E. R. W. Frank, of Berlin, who introduced this treatment into Germany, made a nozzle with an entirely flat point for very sensitive cases with exceedingly small meati.

The irrigator is filled with a warm solution of permanganate of potassium, whose strength will be mentioned later on. The irrigator is then drawn upward by a cord attached to it and passing over a pulley fixed at two metres and a half above the table or chair employed. When the irrigator has reached a height which allows a gentle stream to escape from the nozzle, while the patient, after urinating, sits or lies down, the prepuce, glans, corona, and meatus are carefully cleansed with the solution in the order which they are mentioned. Then the penis is firmly grasped and some of the solution allowed to flow into the meatus and permitted to escape at once. This is repeated, meanwhile gradually raising the irrigator still farther, until successive washings have rendered the entire urethra as clean as possible.

Then the nozzle is held into the meatus, while the patient breathes deeply or makes efforts at urination, until two hundred to five hundred grammes of the solution have flown into the bladder. In most cases the constrictor will be quite readily overcome by the pressure; in others some patience is required. When the bladder is filled, the patient is allowed to exude the fluid, which flows forth in a vigorous stream.

The solution first used is of a strength of 1 part potassium permanganate to 6,000 of warm water; as tolerance is established, this proportion is increased to 1 to 4,000, later to 1 to 2,000, and finally to 1 to 1,000.

These vesical injections are made twice, thrice or four times on the first day; twice on the second, third, fourth, and, if required, on the fifth day, when usually all gonococci have disappeared; then once a day until all discharge has ceased, which in Janet's most severe cases occured on the tenth day. My success has has not been so favorable; I have had several cases in which the flow persisted until the twelfth day.

Before each injection a slide is made for microscopic examination. In the first and second days but little change appears.

Thence forward the gonococci grow sparser in number, and generally on the fourth day at latest they have a swollen appearance; the lumen between each pair of gonococci seems wider, and nowhere can any tendency to further segmentation be observed. They probably are then undergoing a species of involution, or may be returning to that state in which some authors claim them as normal residents of the urethra. On the following day the pus, which has become thin and water-colored, is found free of gonococci.

Then the injections need be given but once a day until the flow ceases entirely. This frequently is on the sixth day of treatment.

It is my habit to order patients to return one week after the flow has ceased. I then make an irritant injection of silver nitrate. A strength of two per centum generally suffices to produce a copious flow within six to twelve hours. If this flow is found to contain gonococci, I repeat the procedures above described until the discharge ceases. Then I allow the patient to rest from treatment for another week. The discharge then produced by silver nitrate contains no gonococci and disappears within twenty-four or forty-eight hours, as do other simple urethrites, without any treatment whatever.

If, however, the discharge evoked by the first silver-nitrate injection shows no gonococci, which occurs in the vast majority of cases, the patient receives another such injection a week later, and if then the discharge is free from gonococci, the patient is discharged, cured.

I hope to demonstrate the technique of this treatment before the summer vacation. I wlll then show that it is a mistake to assume that the bladder can not be filled through the urethra without a catheter. I will also show that no danger whatever, attends forcing gonococci into the bladder with the solution mentioned, and that no complications, beyond an occasional œdema of the entire penis, are incurred by this treatment. I reserve until then also a discussion of the *rationale* of this treatment, for which Prof. Janet can not receive too much credit.—*Correspondence; Journal of Cutaneous and Genito-Urinvry Diseases.*

